# Granulomatous Tubulointerstitial Nephritis as a Rare Cause of Allograft Failure: A Case Report

**DOI:** 10.7759/cureus.76353

**Published:** 2024-12-24

**Authors:** Eugene K Yeboah, Yihe Yang, Okwudili Nnaji, Andrea Roche-Recinos, Subodh Saggi

**Affiliations:** 1 Internal Medicine, State University of New York (SUNY) Downstate Health Sciences University, Brooklyn, USA; 2 Pathology and Laboratory Medicine, Weill Cornell Medicine, New York, USA; 3 Nephrology, State University of New York (SUNY) Downstate Health Sciences University, Brooklyn, USA; 4 Transplant Nephrology, State University of New York (SUNY) Downstate Health Sciences University, Brooklyn, USA

**Keywords:** allograft failure, collapsing focal segmental glomerulosclerosis, collapsing glomerulopathy, focal segmental glomerulosclerosis (fsgs), granulomatous interstitial nephritis

## Abstract

Although granulomatous interstitial nephritis (GIN) is a rare histological finding in kidney transplants, the joint occurrence of GIN and focal segmental glomerulosclerosis (FSGS) has not, to our knowledge, been reported in the literature. We report a case of GIN and de novo FSGS in kidney transplant recipients leading to allograft failure. A 69-year-old male with a history of end-stage renal disease (ESRD) of unknown etiology, as well as liver failure from hepatitis B and C co-infection, initially had a living unrelated kidney transplant (LURT) in 2007 and subsequently received both liver and kidney transplants (SLKTs) in 2017. His second post-transplant course in 2017 was complicated by antibody-mediated rejection, recurrent urinary tract infections, and an episode of cell-mediated rejection, which was treated with both corticosteroids and thyroglobulin. In 2023, his serum creatinine remained stable, ranging from 2.2 to 2.9 mg/dL, with maintenance immunosuppression of mycophenolate mofetil (750 mg twice a day), tacrolimus (4 mg twice a day; target of 5-7 ng/mL), and prednisone (5 mg daily). In February 2024, his serum creatinine increased to 4.3 mg/dL, with nephrotic-range proteinuria (urine protein-to-creatinine ratio of 6000 mg/g) and urinalysis showing pyuria (white blood cell count of 49), but no hematuria. All infectious work-ups, including urine culture, blood culture, QuantiFERON-TB Gold, and sputum *Mycobacterium tuberculosis* polymerase chain reaction (MTB-PCR) (×3), were negative. An allograft biopsy showed acute granulomatous tubulo-interstitial nephritis, along with de novo collapsing FSGS and recurrent diabetic nephropathy. There was no evidence of active T-cell-mediated, vascular, or antibody-mediated rejection (negative C4d). The kidney biopsy for in-tissue MTB-PCR testing was negative. To the best of our knowledge, our patient is the first case of idiopathic GIN with non-necrotizing granulomata, along with de novo collapsing FSGS, leading to allograft failure.

## Introduction

Collapsing focal segmental glomerulosclerosis (FSGS), also known as collapsing glomerulopathy, is defined by the presence of at least one glomerulus with segmental or global wrinkling and retraction of the glomerular basement membranes, associated with hypertrophy and hyperplasia of overlying glomerular epithelial cells [1]. Individually, granulomatous tubulointerstitial nephritis and collapsing FSGS are rarely identified in allograft biopsies [2]. Granulomatous interstitial nephritis (GIN) is found in <1% of allograft biopsies, and up to 60% of the cases are associated with infectious agents: *Mycobacterium tuberculosis*, adenovirus, BK virus, and gram-negative bacteria [3]. Drug hypersensitivity and sarcoidosis are less commonly associated with post-transplant GIN [3]. Our case report highlights the clinicopathologic features of acute granulomatous tubulo-interstitial nephritis, along with de novo collapsing FSGS, leading to allograft failure.

## Case presentation

A 69-year-old male with a past medical history of hypertension, type 2 diabetes mellitus, coronary artery disease (status post percutaneous coronary intervention on aspirin), transcatheter aortic valve replacement, hyperlipidemia, and end-stage renal disease (ESRD) on hemodialysis, as well as liver failure from concomitant hepatitis B and C infection, received a simultaneous liver-kidney transplant (SLKT) in 2017. He previously underwent a living unrelated kidney transplant (LURT) in 2007 in Pakistan, had graft failure in 2012, and became dialysis dependent in 2013. The patient subsequently had an SLKT in 2017, as well as chronic hepatitis B, managed with entecavir, and prior to hepatitis C, treated with Harvoni and ribavirin. He completed treatment for latent tuberculosis with isoniazid in 2016. His second post-transplant course was complicated by recurrent urinary tract infections, antibody-mediated rejection, and, most recently, an episode of cellular-mediated rejection, which was treated with corticosteroids and thymoglobulin. In 2023, his serum creatinine remained stable from 2.2 to 2.9 mg/dL, with maintenance immunosuppression of mycophenolate mofetil (750 mg twice a day), tacrolimus (4 mg twice a day; target of 5-7 ng/mL), and prednisone (5 mg daily). His other home medications were entecavir, aspirin, pravastatin, gabapentin, carvedilol, nifedipine, tamsulosin, and several diabetic management supplies. The patient was a retired NYC sanitation worker, non-smoker, and denied alcohol or drug use.

In February 2024, the patient was referred by a primary care physician to a renal transplant clinic to re-establish care, as he was lost to follow-up and had worsening kidney function. He denied fever, abdominal pain, hematuria, oliguria, abdominal distention, chest pain, nausea, vomiting, and respiratory symptoms. Upon presentation, the patient’s initial vital signs revealed a temperature of 98°F, a heart rate of 61 bpm, and a blood pressure of 119/60 mmHg. The patient’s examination was unremarkable. Initial labs are summarized in Table 1.

**Table 1:** Initial labs IgM: Immunoglobulin M; IgG: Immunoglobulin G; MTB: *Mycobacterium tuberculosis*; PCR: Polymerase chain reaction; DNA: Deoxyribonucleic acid; RNA: Ribonucleic acid

Parameter	Patient Values	Reference Range
Comprehensive Metabolic Panel		
Sodium	137 mmol/L	136-145 mmol/L
Potassium	5.8 mmol/L	3.5-5.1 mmol/L
Calcium	8.0 mg/dL	8.2-10.0 mg/dL
Chloride	111 mmol/L	98-107 mmol/L
Magnesium	1.2 mg/dL	1.9-2.7 mg/dL
Phosphorus	3.8 mg/dL	2.5-5.0 mg/dL
Creatinine	4.3 mg/dL	0.7-1.3 mg/dL
Blood Urea Nitrogen	66 mg/dL	7-25 mg/dL
Carbon Dioxide	15 mmol/L	21-31 mmol/L
Glucose	224 mg/dL	70-99 mg/dL
Anion Gap	17 mmol/L	10-20 mmol/L
Estimated Glomerular Filtration Rate	14 mL/min/1.73 m²	>60 mL/min/1.73 m²
Liver Function Test		
Total Bilirubin	0.3 mg/dL	0.3-1.0 mg/dL
Albumin	4.5 g/dL	3.5-5.7 g/dL
Total Protein	6.9 g/dL	6.0-8.3 g/dL
Aspartate Aminotransferase	6 U/L	13-39 U/L
Alanine Aminotransferase	4 U/L	7-52 U/L
Alkaline Phosphatase	187 U/L	34-104 U/L
Complete Blood Count		
Hemoglobin	8.2 g/dL	14.0-18.0 g/dL
White Blood Count	2.57 k/μL	3.5-10.8 k/μL
Platelet	172 k/μL	130-400 k/μL
Hematocrit	26.8%	42.0-52.0%
Urinalysis		
Blood (Urine)	Negative	Negative
Urine Creatinine	63 mg/dL	20-320 mg/dL
Protein (Urine)	>500 mg/dL	Negative
White Blood Cells (Urine)	49/hpf	<5/hpf
Hyaline Cast	<1/Ipf	<1/Ipf
Protein Spot	>500 mg/dL	<500 mg/dL
Urine Protein Creatinine Ratio	6000 mg/g	<30 mg/g
Coagulation		
Prothrombin Time	12.5 seconds	10.8-13.7 seconds
Activated Partial Thromboplastin Time	30.7 seconds	25-35 seconds
International Normalized Ratio	1.1	<1
Lipid Panel		
Triglyceride	118 mg/dL	0-150 mg/dL
Total Cholesterol	110 mg/dL	0-200 mg/dL
Low Density Lipoprotein Cholesterol	50 mg/dL	<99 mg/dL
High Density Lipoprotein Cholesterol	36 mg/dL	30-85 mg/dL
Anemia Work-Up		
Iron	32 μg/dL	50-212 μg/dL
Total Iron Binding Capacity	305 μg/dL	240-450 μg/dL
Unsaturated Iron Binding Capacity	273 μg/dL	155-355 μg/dL
Ferritin	839.7ng/mL	16.0-294.0 ng/mL
Mineral Bone Disease		
Vitamin D	45.3 pg/mL	18-72 pg/mL
Parathyroid Hormone	424.4 pg/mL	15.0-65.0 pg/mL
Hemoglobin A1C	7.4%	<5.7%
Infectious Work-Up		
COVID-19	Negative	Negative
Influenza Virus	Negative	Negative
HIV 1/2 Ag/Ab	Negative	Negative
Hepatitis A Virus	Non-reactive	Non-reactive
Hepatitis B Surface Antigen	Non-reactive	Non-reactive
Hepatitis B Core IgM	Reactive	Non-reactive
Hepatitis B e-Antigen	Non-reactive	Non-reactive
Hepatitis B Surface Antibodies	Negative	Negative
Hepatitis B Viral Load	Not detectable	Negative RNA copies/mL
Cytomegalovirus DNA	Negative	Negative
Cytomegalovirus Quantitative PCR	2.61	Negative
Hepatitis C IgG	Negative	Negative
QuantiFERON Tuberculosis Gold	Negative	Negative
Sputum MTB-PCR	Negative (x3)	Negative
Kidney Tissue MTB-PCR	Negative	Negative

Imaging

**Figure 1:**
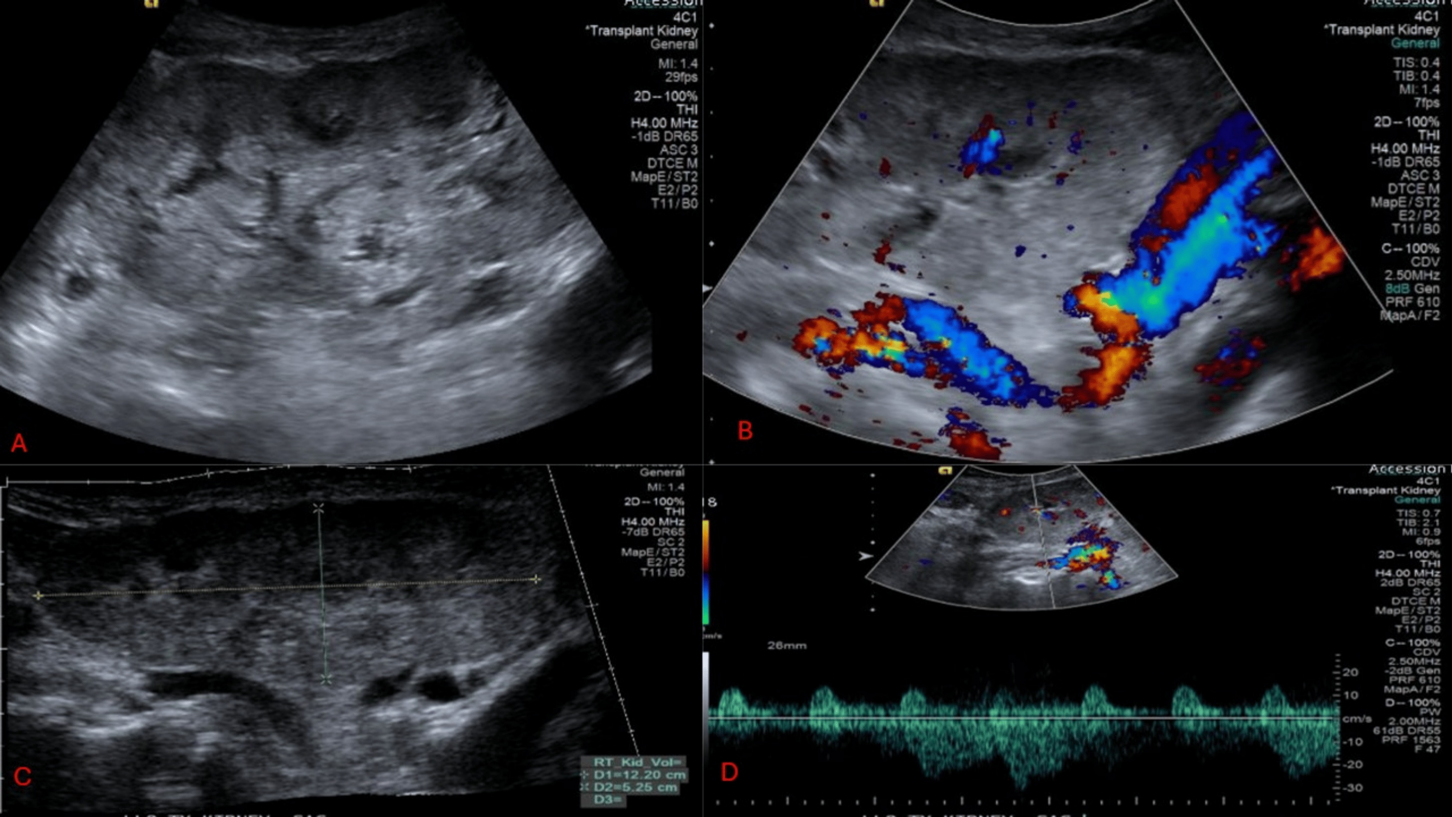
Transplanted kidney ultrasound (A) There is increased echogenicity, but normal parenchymal thickness and contour, no calculi, no pelvicalyceal dilation, no cysts, and no solid masses, with no peri-transplant fluid collection; (B) Doppler ultrasound of transplanted kidney; (C) Transplanted kidney size: 12.2 x 5.3 x 5.9 cm; (D) Transplanted kidney resistive indices: 0.64, 0.61, 0.60 at the upper, middle, and lower poles, respectively (normally less than 0.7).

Figure 1 shows a kidney ultrasound done on March 18, 2024. The renal allograft is visualized in the right lower quadrant. There is increased echogenicity, but normal parenchymal thickness and contour, with no calculi, no pelvicalyceal dilation, no cysts, and no solid masses, and no peri-transplant fluid collection. The transplanted kidney size is 12.2 x 5.3 x 5.9 cm, with resistive indices of 0.64, 0.61, and 0.60 at the upper, middle, and lower poles, respectively (normally less than 0.7).

Kidney biopsy

**Figure 2:**
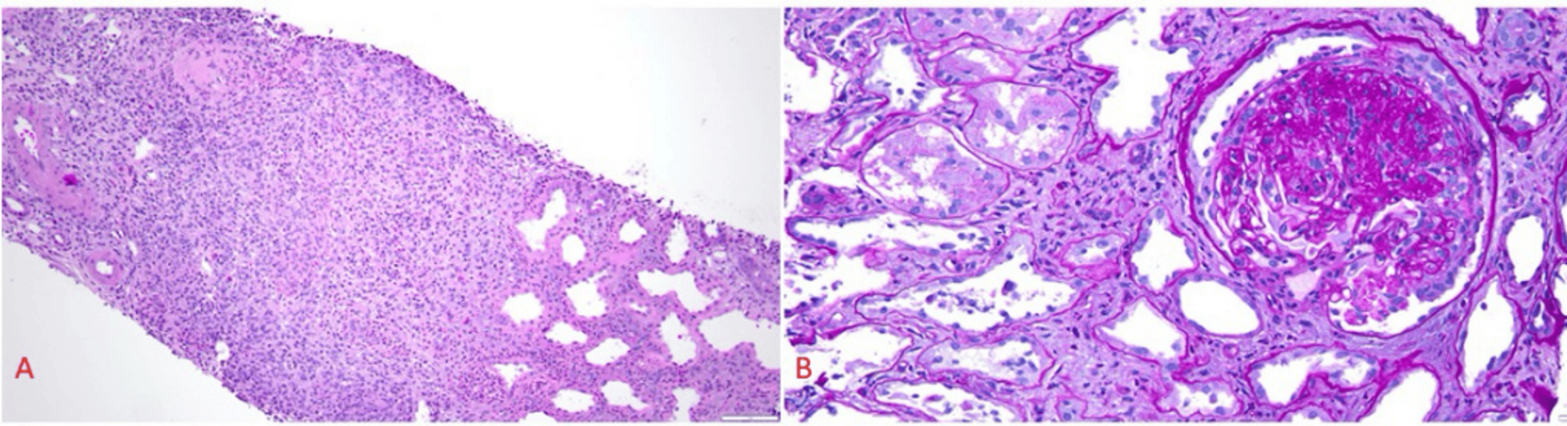
Transplanted kidney biopsy (A) This figure shows severe acute neutrophil-rich tubulointerstitial inflammation, numerous neutrophil casts (pus casts), and non-caseating granulomata (H&E stain, 40x); (B) This figure shows focal and segmental glomerulosclerosis with collapsing features, with no features of rejection, moderate to severe tubular atrophy, mild interstitial fibrosis, as well as severe arterial and arteriolar sclerosis, and focal arteriolar hyalinosis (PAS stain, 200x). H&E: Hematoxylin and eosin; PAS: Periodic acid-Schiff

Figure 2 shows a transplanted kidney biopsy done on April 10, 2024. The biopsy showed acute pyelonephritis, with severe acute neutrophil-rich tubulointerstitial inflammation, numerous neutrophil casts (pus casts), and non-necrotizing granulomata. It also showed evidence of recurrent diabetic kidney disease, with no features of rejection, moderate to severe tubular atrophy, mild interstitial fibrosis, severe arterial and arteriolar sclerosis, and focal arteriolar hyalinosis.

Later, in 2024, the patient was restarted on intermittent hemodialysis, given declining allograft function despite starting all immunosuppressive therapy, including steroids.

## Discussion

GIN is an uncommon histologic diagnosis that is present in <1% of native renal biopsies [2]. According to Meehan et al. [4], the incidence of GIN in renal allograft biopsies was 0.6%. GIN was identified in 0.3% of biopsies, at a mean of 552 days post-transplantation [5]. Collapsing FSGS, also known as collapsing glomerulopathy, is defined by the presence of at least one glomerulus with segmental or global wrinkling and retraction of the glomerular basement membranes, accompanied by hypertrophy and hyperplasia of overlying glomerular epithelial cells [6]. Collapsing FSGS in the native kidney is associated with heavy proteinuria and accelerated renal failure [6]. However, collapsing FSGS in the renal allograft is less well described [6].

Acute rejection, which is characterized by infiltrating T cells and macrophages in allografts, is the most common cause of tubulointerstitial nephritis in renal allografts [2]. However, some studies showed that in the majority of cases of GIN in renal allografts, infection is presumed to be the main cause, which is different from the causes of GIN in native kidneys [2]. The reason for the difference is presumed to be that transplant recipients receive immunosuppression therapy; hence, their autoinflammatory responses are repressed [2]. In native kidneys, some studies have documented risk factors associated with GIN. In one study involving 12 patients, sarcoidosis, medication, and infection accounted for three cases each [2]. One each of the remaining three GIN cases was associated with granulomatosis polyangiitis and oxalosis, respectively, whereas the last patient was presumed to have idiopathic GIN [2]. In other studies, sarcoidosis has been described as a cause of GIN [2,7,8], although some authors consider this entity to be a case of isolated renal sarcoidosis [2,8]. In another study involving 18 cases of GIN, five cases were associated with sarcoidosis, whereas two were associated with tubulointerstitial nephritis and uveitis, two with drugs, and nine were idiopathic [2,9]. 

Donor’s APOL-1 high-risk variants have been proposed to increase the risk of de novo collapsing FSGS, particularly in recipients with additional injury induced by viruses or bacterial infections [10]. De novo post-transplant collapsing FSGS has been reported in 10-20%, associated with hyperfiltration injury, chronic transplant glomerulopathy, and calcineurin inhibitor toxicity [11]. Our case report, however, showed acute pyelonephritis, with severe acute neutrophil-rich tubulointerstitial inflammation, numerous neutrophil casts (pus casts), and non-necrotizing granulomata, along with de novo collapsing FSGS and recurrent diabetic nephropathy. An extensive workup was done for non-necrotizing granuloma, which was negative. To our knowledge, there have been no prior reports of idiopathic GIN associated with non-necrotizing granulomata, along with de novo collapsing FSGS in renal allografts.

In one study involving seven patients who received prednisolone therapy, renal function improved in five of the seven patients [2]. Joss et al. [9] also showed that steroids were efficacious for idiopathic GIN. Another study described a good response following treatment with an antibody to tumor necrosis factor-alpha [2,12]. All these reported cases showed the efficacy of steroids in the management of GIN. In our report, our patient had a biopsy-documented GIN, along with a non-necrotizing granuloma and de novo collapsing FSGS, and received both steroids and other immunosuppressives. Despite these medications, our patient’s kidney function still deteriorated, requiring dialysis eventually.

## Conclusions

Infection is the main causative factor of GIN in renal allografts. We reported acute granulomatous tubulointerstitial nephritis, along with de novo collapsing FSGS, as a rare cause of allograft failure. The risk factors associated with this uncommon form of glomerular injury in kidney allografts include, but are not limited to, the donor's high-risk APOL1 genotype, viral infections (such as cytomegalovirus, human immunodeficiency virus, Epstein-Barr virus, SARS-CoV-2, parvovirus, and BK virus). The significance of idiopathic GIN in allografts remains unknown, as our patient’s kidney function deteriorated despite immunosuppressive therapy. Therefore, it is necessary to collect more data on this condition.
